# Imaging, Histopathologic, and Treatment Nuances of Pulmonary Carcinosarcoma

**DOI:** 10.1155/2017/8135957

**Published:** 2017-09-17

**Authors:** Tyler Gleason, Michael Haas, Brian H. Le

**Affiliations:** ^1^Department of Medicine, Reading Hospital, West Reading, PA 19611, USA; ^2^Department of Radiology and Radiation Oncology, Reading Hospital, West Reading, PA 19611, USA; ^3^Department of Pathology, Reading Hospital, West Reading, PA 19611, USA

## Abstract

A 76-year-old female with coronary artery disease, chronic obstructive pulmonary disease, diabetes mellitus type II, and 40 pack-year smoking history presented with a four-day history of cough, productive of green-yellow sputum. Chest X-ray revealed opacification of the left upper lung field, and computed tomography (CT) of the chest showed a large cavitary lesion invading the T2-T3 vertebral bodies, extending into the epidural space, giving rise to mild cord compression. Biopsy of the lesion revealed a poorly differentiated neoplasm composed of distinct epithelial and mesenchymal components, consistent with carcinosarcoma. A metastatic workup was negative. Primary lung carcinosarcoma is a rare tumour that can demonstrate an especially aggressive clinical course; diagnosis is often nuanced by limited sampling at initial presentation, especially in a setting of advanced disease and debility that precludes consideration for upfront resection or more extensive, invasive sampling.

## 1. Introduction

Primary lung carcinosarcoma is a rare tumour that can demonstrate an aggressive clinical course. The diagnosis is often complicated by a lack of definitive, characteristic imaging findings; the heterogeneity of the tumour itself may give rise to a wide list of differential diagnostic considerations, and biopsies reflecting a small sampling of the lesion may fail to capture both the epithelial and sarcomatous features of the tumour. Prompt and accurate diagnosis is essential, as these tumours can often progress from asymptomatic to life threatening over a short time-course, as illustrated in the following case.

## 2. Case Presentation

A 76-year-old female with a history of coronary artery disease, chronic obstructive pulmonary disease, and diabetes mellitus type II presented to the emergency room with a chief complaint of cough, productive of green-yellow sputum over four days. She was a former smoker with a 40 pack-year history but transitioned to electronic cigarettes eight months prior. She denied any fever, chills, or night sweats but did indicate having some upper back pain and a 10–15-pound weight loss over the previous three months. On physical exam, there was normal auscultation and percussion of the lungs bilaterally. Cardiac, abdominal, and all other system exams were normal. Specifically, the complete neurologic exam was unremarkable, unrevealing of any focal deficits.

**Figure 1:**
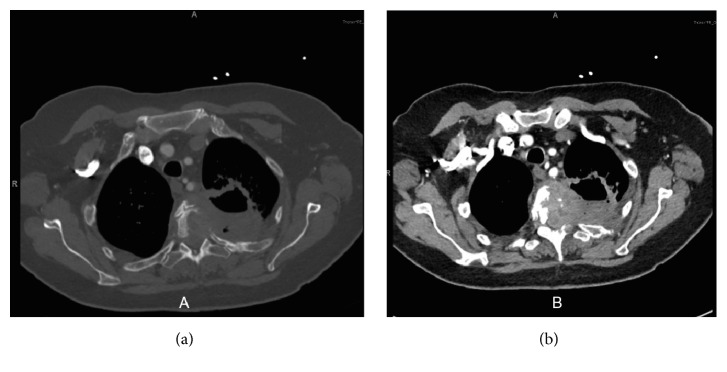
Computed tomography (CT) of the chest in the axial plane with (a) bone window and (b) soft tissue window.

**Figure 2:**
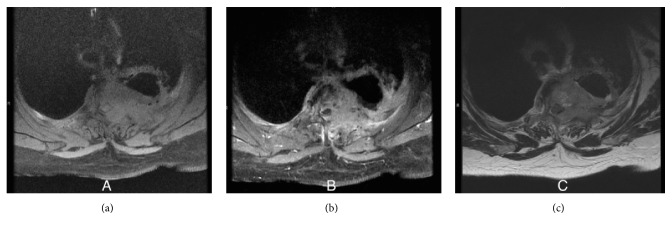
Magnetic resonance imaging (MRI) of the thoracic spine in the axial plane with (a) T1-weighted sequence without contrast, (b) T1-weighted sequence with contrast, and (c) T2-weighted sequence.

**Figure 3:**
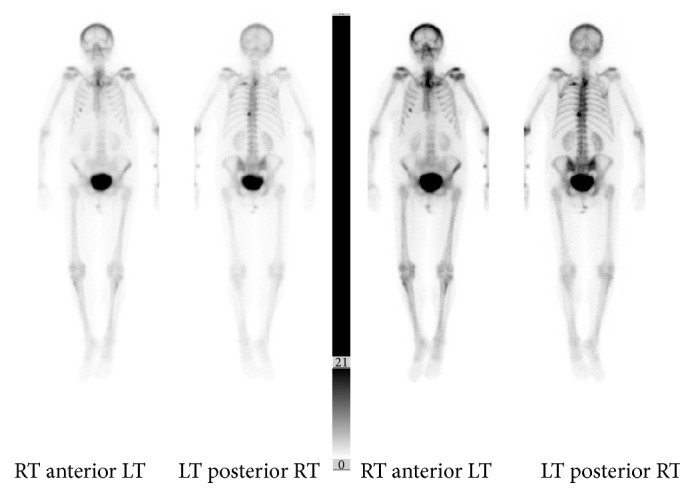
Bone scan showing tracer uptake in the upper T-spine and left 4th rib, corresponding to areas of direct tumour involvement.

**Figure 4:**
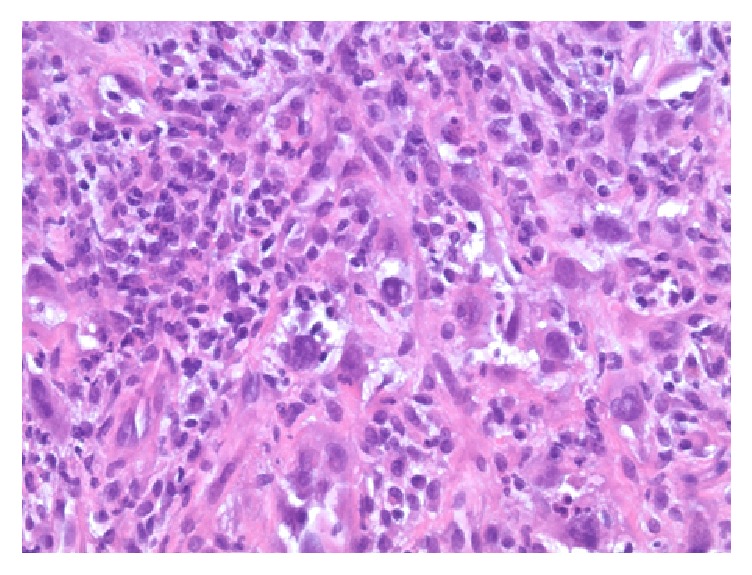
Intermediate power view of the tumour showing a cellular proliferation of pleomorphic cells with epithelioid morphology (H&E stain, 200x original magnification).

**Figure 5:**
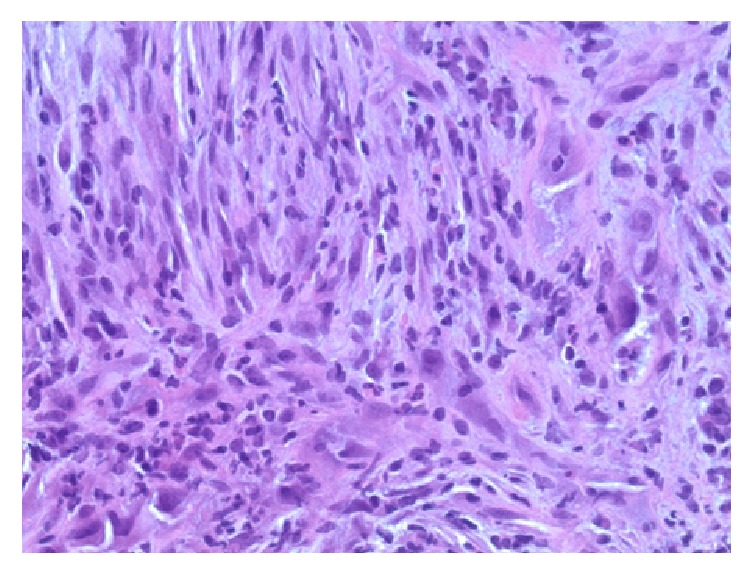
Intermediate power view of the tumour reflecting the spindled, sarcomatoid component (H&E stain, 200x original magnification).

**Figure 6:**
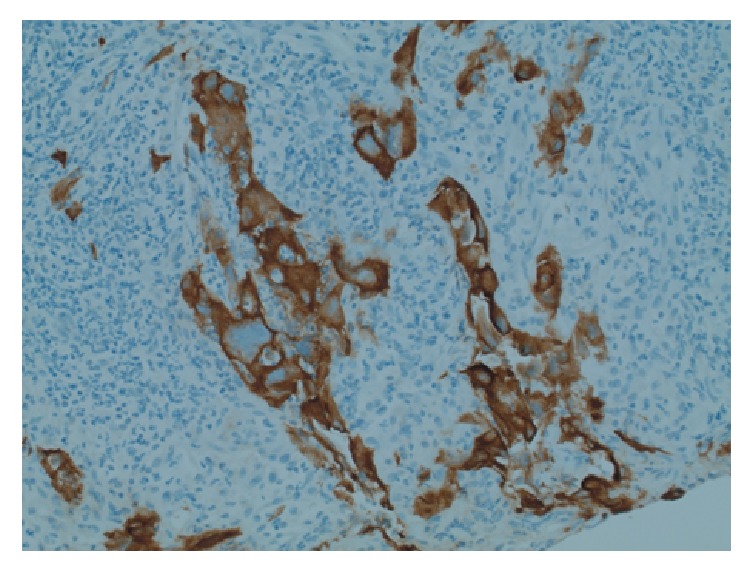
Pan-cytokeratin immunohistochemistry, accentuating the epithelial component within the tumour (200x original magnification).

**Figure 7:**
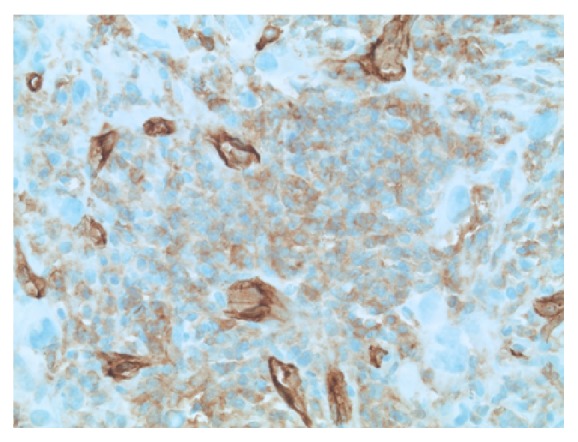
Smooth muscle actin immunohistochemistry, demonstrating reactivity in the mesenchymal component (200x original magnification).

Initial chest X-ray showed a cavitary lesion in the left upper lung field. Subsequent computed tomography (CT) scan of the chest with contrast revealed a cavitary, bilobed, thick-walled mass measuring 6.0 × 6.0 × 8.0 centimetres (cm) in the left upper lobe, invading into the posterior mediastinum with confluent involvement of the T2 and T3 vertebral bodies. This was accompanied by significant bony destruction and invasion into the spinal canal. Magnetic resonance imaging (MRI) of the T-spine was obtained to further characterize the degree of cord compression and showed the same infiltrating mass filling the left T2-T3 neural foramen (Figures 1 and 2). A bone scan was notable for tracer uptake in the left 4th rib and T2-T3 spine, correlating with direct tumour involvement (Figure 3). Brain MRI with and without contrast showed no evidence of metastasis. Fluorodeoxyglucose (FDG) PET-CT had not been performed at this point. In an attempt to characterize the tumour, five CT-guided core biopsies were obtained by interventional radiology and sent for pathologic examination. Histologic examination showed a proliferation of markedly pleomorphic cells, with some demonstrating epithelioid morphology (Figure 4), interlaced with cellular regions showing a spindled, sarcomatoid appearance (Figure 5). Pan-cytokeratin and other epithelial markers accentuated the distinct epithelial component (Figure 6), while smooth muscle actin and CD31 immunohistochemistry highlighted the spindled, sarcomatoid regions (Figure 7). These findings indicated a biphasic, mixed epithelial-mesenchymal neoplasm, consistent with carcinosarcoma. To complete the metastatic workup, CT of the abdomen and pelvis and an MRI of the brain were performed, showing no evidence of metastatic disease, thus supporting a diagnosis of primary lung carcinosarcoma.

The neurosurgical team evaluated the patient and did not feel there was an emergent indication for surgery to address tumour invasion into the thoracic vertebral bodies and spinal canal. Radiation oncology and medical oncology also evaluated the patient and felt that there was no indication for immediate radiation therapy for cord compression; nevertheless, as the patient was neurologically intact, the interdisciplinary team concluded that it would be prudent to perform a CT simulation immediately in case the need for emergent radiation therapy arose. The day after CT simulation occurred, the patient fell while attempting to ambulate. Repeat neurological exam elicited significant weakness and hyperreflexia in the lower extremities bilaterally. Intravenous dexamethasone was initiated (4 mg, three times a day) and the patient received 3 gray (Gy) of radiation each day for the following two days. As symptoms did not improve after two days of treatment, dexamethasone dosage and frequency were increased (6 mg every six hours), and radiation therapy was extended for a total of 30 Gy in ten fractions to the thoracic spine.

After five days of treatment, the patient was discharged to a skilled nursing facility, with plans to continue the 10-day course of radiation therapy, to be followed by a transition to outpatient hospice. However, the patient returned to the hospital with acute respiratory failure two days following discharge. At that time, the decision was made by the patient and her family to forgo further palliative radiation and transition to inpatient hospice care. The patient passed away on day four of inpatient hospice, only 15 days after first presenting to the emergency room with cough and sputum production.

## 3. Discussion

The differential diagnosis for a cavitary lung lesion is broad and can primarily be triaged into infectious and noninfectious aetiologies [1]. Among infectious causes, the most common pathogens causing cavitary lesions include bacteria (*Klebsiella* and* Staph aureus*), mycobacterial (tuberculosis, avium complex), fungi (*Aspergillus*,* Blastomyces*,* Coccidioides*, and histoplasma), and parasite (*Echinococcus*). The most common noninfectious aetiologies are malignancies, reflecting either primary lung malignancies or metastases. Other differential diagnostic considerations include rheumatologic diseases, such as granulomatosis with polyangiitis and sarcoidosis. There are also various other aetiologies for cavitary lesions that are much less frequently encountered, including pulmonary embolism and cryptogenic organizing pneumonia [1]. Differentiating between the various causes of cavitary lesions is often difficult and requires a thorough evaluation of the clinical, laboratory, and imaging data. Certain imaging variables, such as wall thickness of the cavity and multiple enlarged mediastinal lymph nodes, have been proposed as diagnostic factors in discriminating between malignant and nonmalignant aetiologies but are imperfect at best and remain controversial [2–4]. The diagnostic picture is often complicated by the possible coexistence of other malignancies and infectious processes [1]. Ultimately, a definitive diagnosis often relies on biopsy and pathologic evaluation.

Greater than 95% of all primary lung cancers fall into one of four cell types: adenocarcinoma, squamous cell carcinoma, undifferentiated large cell carcinoma, and small cell carcinoma [5]. Adenocarcinoma and undifferentiated large cell carcinoma typically present radiographically as solitary peripheral nodules, while squamous and small cell carcinomas tend to be more central and may manifest as hilar masses, giving rise to atelectasis or obstructive pneumonia. Prognosis is poor for all primary lung cancers but is in general the best for squamous cell carcinoma and worst for small cell carcinoma, with adenocarcinoma and undifferentiated large cell carcinoma having intermediate prognoses [5]. Among the primary lung cancers, roughly 20% will show cavitation on chest CT, with squamous cell carcinoma showing the highest proclivity for cavitation [1]. In fact, cavitation is generally associated with an especially poor prognosis [6]. Unfortunately, there are no definitive imaging findings to differentiate among the four most common causes of primary lung cancer. Therefore, biopsy and pathologic analysis of an unknown lung mass is almost always indicated, either by bronchoscopy for central lesions or by CT-guided biopsy for peripheral lesions, to approach a more definitive diagnosis that would guide further management.

Primary lung carcinosarcoma is a rare tumour, representing roughly 0.1–1.0% of all lung cancers [7–9]. These tumours show a roughly 7 : 1 male-to-female ratio, present at a median age of 65 years, and are frequently associated with a history of heavy smoking. Pulmonary carcinosarcoma was first described in the literature in 1908 but remains poorly understood due to their rarity and complex features. A 2004 classification by the World Health Organization (WHO) grouped sarcomatoid carcinomas into several categories, including pleomorphic carcinoma, spindle cell carcinoma, giant cell carcinoma, carcinosarcoma, and pulmonary blastoma [10]. These tumours have significant overlap and share many common features, but there are enough histological differences to suggest that they are separate entities [11].

Several theories exist concerning the pathogenesis of carcinosarcoma. One proposes that the neoplasm is essentially a collision tumour, arising from a separate epithelial precursor and a distinct mesenchymal component. Another postulate is that the tumour arises from either an epithelial or mesenchymal cell line and subsequently undergoes metaplastic transformation. Finally, a third theory suggests that the lesion arises from a multipotent cell with potential for both, mesenchymal and epithelial differentiation [8]. Currently, the second and third theories are favoured over the first, but there is still considerable disagreement in the literature [12].

Of the primary lung carcinosarcoma cases reported in the literature, this is the first to our knowledge that demonstrates direct extension into the spinal canal, with extensive destruction of the vertebral bodies and compression of the spinal cord, ultimately leading to paralysis in the lower extremities for our patient. One hallmark of these tumours is that they are highly aggressive and have poor outcomes, with one case series demonstrating 5-year survival rate of 21.3% even though 60% of the patients had stage 1 disease at time of diagnosis. Notably, unlike in small cell carcinoma, tumour size was related to survival. Patients with tumours less than 6 cm had a 40% 5-year survival, while patients with tumours greater than 6 centimetres had a 10% 5-year survival [11].

The diagnosis in this scenario was especially nuanced by the fact that only a limited amount of tissue could be obtained for diagnosis. As the extent of tumour and the degree of patient debility at the time of presentation precluded an aggressive surgical procedure, needle biopsy was deemed as the most viable option for lesional sampling. Although a full immunohistochemical and molecular workup would be ideal, such was not feasible in consideration of the limited material obtained. Nevertheless, a diagnosis of pulmonary carcinosarcoma was rendered in demonstration of distinct epithelial and mesenchymal neoplastic components on tissue examination and by exclusion of other possible primary sites by imaging.

As the prognosis of pulmonary carcinosarcoma is generally poor, when diagnosed, an upfront discussion regarding expectations of outcome would be prudent, as was the case in this scenario.
